# Adiponectin Suppresses UVB-Induced Premature Senescence and hBD2 Overexpression in Human Keratinocytes

**DOI:** 10.1371/journal.pone.0161247

**Published:** 2016-08-15

**Authors:** MinJeong Kim, Kui Young Park, Mi-Kyung Lee, Taewon Jin, Seong Jun Seo

**Affiliations:** 1 Departments of Dermatology, Chung-Ang University Hospital, Chung-Ang University College of Medicine, Seoul, South Korea; 2 Department of Laboratory Medicine, Chung-Ang University Hospital, Chung-Ang University College of Medicine, Seoul, Korea; San Gallicano Dermatologic Institute, ITALY

## Abstract

Recent studies have revealed that adiponectin can suppress cellular inflammatory signaling pathways. This study aimed to elucidate the effect of adiponectin on the unregulated production of hBD2 in UVB-induced premature senescent keratinocytes. We constructed an *in vitro* model of premature senescent keratinocytes through repeated exposure to low energy UVB. After repeated low energy UVB exposure, there was significant generation of reactive oxygen species (ROS) and induction of senescence-associated markers, including senescence associated beta-galactosidase activity and expression of p16^INK4a^ and histone H2AX. In addition, the present clinical study showed higher expression of hBD2 in sun-exposed skin of elderly group, and the overexpression of hBD2 was observed by c-Fos activation *in vitro*. Adiponectin has the ability to scavenge ROS and consequently inhibit MAPKs and SA-markers in UVB-exposed keratinocytes. An inhibitor study demonstrated that adiponectin downregulated hBD2 mRNA expression through suppression of the AP-1 transcription factor components c-Fos via inactivation of p38 MAPK. Collectively, the dysregulated production of hBD2 by the induction of oxidative stress was attenuated by adiponectin through the suppression of p38 and JNK/SAPK MAPK signaling in UVB-mediated premature senescent inducible conditions. These results suggest the feasibility of adiponectin as an anti-photoaging and anti-inflammatory agent in the skin.

## Introduction

As the skin is the most exposed tissue to environmental stress, repeated exposure to environmental stimuli include ultraviolet (UV) light leads to an enhanced inflammatory state and even aging of skin and a reduction of barrier function [1, 2]. An essential component of the cutaneous defensive barrier, human beta defensin 2 (hBD2) is a cysteine-rich cationic 41 amino acid antimicrobial peptide, which is induced in cutaneous infection and inflammatory dermatitis [3]. It plays a role not only in cellular immune reactions and antimicrobial cytotoxic activities against bacteria, fungi, and viruses but also as a sacrificial sunscreens in UV exposed tissue [4, 5]. Moreover, as a chemoattractant for immature dendritic cells, memory helper T cells, monocytes, macrophages, and activated neutrophils, beta defensin provides a link between the innate and adaptive responses against microbial infection [6–9]. Recently, reports showed that the overexpression of hBD2 stimulated epidermal keratinocyte migration and proliferation, and even played an oncogenic role in esophageal carcinogenesis and basal cell carcinoma [3, 10, 11], and could also result in excessive inflammation similar to that seen in psoriasis and rosacea [12, 13]. Furthermore, clinical studies reported that high hBD2 levels were found in elderly individuals and UV exposed condition, possibly due to age-dependent skin changes, including barrier disruption, that were caused by an accumulated inflammatory response [2, 14]. Therefore, loss of hBD2 homeostasis may mediate the excessive inflammatory responses and the correlation between photoaging and hBD2 expression is hypothesized in this study.

UV light is a known hBD2 inducer [15, 16] that is mediated by pro-inflammatory signal transduction mostly dependent on mitogen activated protein kinases (MAPKs); particularly the p38, Jun amino-terminal kinase (JNK)/stress-activated protein kinases (SAPK) and Extracellular signal-regulated kinases (ERK)) [17, 18]. Indeed, the hBD2 promoter contains the activator protein 1 (AP-1)-binding sequences [19]. AP-1 is an important regulatory transcription factor that contributes to the inflammatory and immune responses. AP-1 is a protein dimer that consists of Fos (c-Fos, Fra-1, Fra-2) and Jun (c-Jun, JunB, JunD) family members, and its expression is regulated via MAPKs in UV-exposed keratinocytes [20].

Especially, UVB induced reactive oxygen species (ROS) production critically impacts DNA damage and increases oxidative stress, with the consequent activation of MAPK signaling pathways [21]. To protect the skin from UV-induced damage, the skin has efficient DNA repair systems and tumor suppressor mechanisms to prevent the growth of cells at risk of neoplastic transformation, so-called “cellular senescence” [22, 23]. These senescent cells show specific morphological and functional changes and senescence-associated beta-galactosidase (SA-β-gal) activity and high expression of several senescence-associated markers such as p16^INK4a^ and histone H2AX [24–27]. However, senescent cells have negative aspects that might contribute to alterations in tissue structure or function leading to skin aging [28–30].

Recently, several observations suggested a potential role of adiponectin, a known adipokine that circulates in high concentrations accounting for 0.01% of total serum protein [31, 32], as an anti-inflammatory plasma protein [33–35], and the function of adiponectin has received attention dermatology, including studies in cutaneous wound healing, skin sensitivity, and psoriasis [36–38]. However, the role and mechanism of adiponectin in inflammatory response-mediated premature senescence of keratinocytes remains unclear. In this study, we suggest that there is the correlation between photoaging and hBD2 expression and the adiponectin plays a role in protective effect against hBD2 dysregulation in UVB-induced premature senescent keratinocytes.

## Materials and Methods

### Chemicals and preparation

Normal human epidermal keratinocytes (NHEK) were purchased from Gibco BRL, Life Technologies (Grand Island, NY, USA). Cell culture medium [EpiLife, with calcium], Human Keratinocyte Growth Serum, and other materials required for culturing cells were purchased from Gibco BRL, Life Technologies. Recombinant human adiponectin, produced in *E*. *coli*, having a molecular weight of 25.1 kDa and containing 231 amino acids (15–244) was purchased from BioBud (Sung-Nam, Gyeong-Gi, Korea). A SA-β-galactosidase staining kit and specific antibodies used for Western blot analysis were purchased from Cell signaling technology (Danvers, MA, USA). 3-(4,5-dimethylthiazol-2-yl)-2,5-diphenyltetrazolium bromide (MTT) was acquired from Sigma (St. Louis, MO, USA). The pharmacologic inhibitors of JNK (SP600125), and p38 (SB203580) were purchased from Calbiochem (San Diego, CA, USA). Other chemicals and reagents used in this study were of analytical grade.

### Repeated UVB irradiation

For UVB exposure studies, we use two TL 20 W/12 RS lamps (Philips, Eindhoven, Netherlands), emitting UVB peaking at 313 nm that were placed 15 cm above the cell culture flasks. The emitted radiation was checked at the bottom of each flask using a UV-meter (Waldmann, Villingen-Schwenningen, Germany). For repeated exposures to UVB, keratinocytes were seeded in flasks 2 days before and the cells were exposed to UVB 5 mJ/cm^2^ in a serum free medium without flask cover. This exposure was repeated 6 times and the time interval between exposures was 30 min. Normal control (NC) cells were submitted to the same conditions but with no exposure to UVB.

### Cell culture and viability assay

NHEK were maintained in a 5% CO_2_ humidified atmosphere at 37 in EpiLife supplemented with calcium. The cells were used at the second to third passages. Before the experiment, the cells were conditioned 4 to 6 h in serum-free medium. Cell viability was determined by MTT reduction assays, as described by Hansen, Nielsen [39]. In brief, the cells were incubated for at least 24 h in 96-well plates and pre-treated with 10 μg/mL concentrations of adiponectin for 24 h. Then, the cells were treated with repeated UVB exposures at room temperature. After the culture supernatants were removed, the resulting purple formazan was dissolved with dimethyl sulfoxide (DMSO) [40]. Absorbance values were read at 562 nm using a microplate spectrophotometer, SpectraMax340pc (Molecular Devices, Sunnyvale, CA, USA). Cell viability was calculated relative to the absorbance of the UVB untreated NC group.

### Assay for senescence-associated beta-galactosidase (SA-β-gal) activity

SA-β-gal activity was determined as described by Debacq-Chainiaux *et al*. [41]. In brief, the cells were washed twice with phosphate-buffered saline (PBS) and fixed with 2% formaldehyde/0.2% glutaraldehyde at room temperature for 10 min. After two additional washes with PBS, 1 ml of staining solution (40 mM citric acid/Na phosphate buffer, 5 mM K_4_[Fe(CN)_6_]_3_∙H_2_O, 5 mM K_3_[Fe(CN)_6_], 150 mM sodium chloride, 2 mM magnesium chloride and 1 mg/mL X-gal in distilled water, pH 6.0) were added to the cells, and incubated overnight at 37°C. The degree of cell senescence was quantified as the percentage of SA-β-gal positive cells and expressed as a percentage of the NC.

### Western blot analysis

Standard procedures were used for Western blotting. Briefly, the cells were lysed in 1% Triton-X radioimmunoprecipitation assay (RIPA) buffer for 1 min. The remaining cell debris was removed by centrifugation. The protein concentrations of the cell lysates were determined using the bicinchoninic acid assay (BCA) method. Cell lysates containing equal amounts of protein (~ 35 μg of total protein) were separated by sodium dodecyl sulphate-polyacrylamide gel electrophoresis (SDS-PAGE) and electro-blotted onto polyvinylidene difluoride (PVDF) membranes. The membranes were blocked with 5% bovine serum albumin (BSA) and then incubated with desired primary and secondary antibodies. Protein expression was detected using the EzWestLumi plus system (ATTO, Tokyo, Japan), according to the manufacturer’s instructions. Blots were visualized using a ChemiDoc^TM^ XRS image analyzer (Bio-Rad, Hercules, CA, USA), and the protein expression levels were quantified using ImageJ software and normalized to GAPDH and β-actin.

### Intracellular ROS assay

Intracellular ROS levels were measured by detecting fluorescence intensity of the oxidant-sensitive 2’,7’-dichloro-dihydro-fluorescein diacetate (DCFH-DA). In brief, DCFH-DA diffuses into cells and is de-acetylated by cellular esterases to non-fluorescent DCFH, which is rapidly oxidized to highly fluorescent 2’,7’-dichlorodihydrofluorescein (DCF) by ROS. Human keratinocytes grown in fluorescence microtiter 96-well black plates were pre-incubated for at least 24 h and incubated with adiponectin (10 μg/ml) for an additional 24 h. The conditioned cells were washed once with PBS, labeled with 20 uM DCFH-DA in serum free media, and incubated for 30 min in the dark at 37°C. Then, UVB exposure was repeated 6 times and the time interval between exposures was 30 min. After the cells were washed once with PBS, intensity of the fluorescence signal was detected with an excitation wavelength of 485 nm and emission wavelength of 535 nm using a SpectraMax i3x microplate reader (Molecular Devices). The relative induction of ROS was normalized by subsequent staining and measurement of PI (propidium iodide) fluorescence.

### RNA extraction and quantitative reverse transcription (RT)-PCR analysis

Total RNA was extracted from keratinocytes using TRIzol® reagent (Welgene, Seoul, Korea) as reported in the manufacturer’s manual. The cDNA synthesized from mRNA was then incubated for an additional 1 h at 42°C. Single-stranded cDNA was amplified by PCR with specific primers for genes encodinghBD2, c-Jun, c-Fos, and GAPDH. Quantification of mRNA was performed using a real-time thermal cycler (Bio-Rad) using SYBR premix Ex Taq (Takara, Shiga, Japan), as reported in the manufacturer’s manual. All reactions were carried out in duplicate and the results were analyzed via the 2^-ΔΔCT^ method and data were normalized to GAPDH.

### Immunofluorescence

Cells were fixed with 4% paraformaldehyde in PBS and permeabilized with 0.5% BSA, 0.2% Triton X-100 in PBS. Cells were incubated in 5% BSA blocking solution, washed in PBS, and incubated overnight with anti-human beta defensin 2 goat IgG (sc-10854, clone C-17, Santa Cruz Biotechnology). Secondary antibody was administered for 1 h and nuclei were stained by 4′,6-diamidino-2-phenylindole (DAPI) (mounting medium with DAPI (Abcam, Cambridge, MA, USA)).

### Quantification of human beta defensin 2 from human-derived corneocytes

The Institutional Review Board (IRB) of the Chung-Ang University hospital (Seoul, Korea) (IRB No. C2015210) approved the protocol for the collection of human-derived corneocytes. Forty-two healthy individuals were recruited, 21 individuals from 20 to 29 years of age (young adults) and 21 individuals from 64 to 81 years of age (elderly adults). All participants had no autoimmune diseases, cancer, diabetes, or dermatitis (either atopic, psoriasis, eczema, contact, or seborrheic) and did not use immunosuppressive drugs for at least 6 months. To obtain human skin corneocytes, 10 sheets of D-squame discs (a diameter of 22 mm, CuDerm Corp., Dallas, TX, USA) were applied to both the abdomen (sun protected) and dorsal hand (sun exposed) areas. The acquired D-squame discs were stored in a 20 ml glass bottle in a -80°C deep freezer until use. Proteins were extracted from the acquired D-squame discs using the Qproteome Mammalian Protein Prep kit (Qiagen, Hilden, Germany) according to the manufacturer’s instructions. The concentration of acquired proteins was determined by Bradford assay. The expression level of hBD2 in the corneocytes was determined with a human beta defensin 2 ELISA kit (Peprotech, Cedarlane, Burlington, ON, Canada). The concentration of beta defensin 2 total protein was determined by comparing the optical density (O.D.) of the samples to the standard curve. The measurements were carried out in duplicate.

### Statistical analysis

All *in vitro* data are presented as the mean ± standard error (SE). The mean values were calculated based on data from at least three independent experiments that were conducted on separate days using freshly prepared reagents. Data were analyzed using the paired t-test. Clinical characteristics data were analyzed using non-parametric multiple comparison Kruskal-Wallis tests with a post hoc Mann-Whitney test to identify differences between groups (n = 21). The significance of differences was defined at a *P* value < 0.05 and the statistical analysis was performed using SPSS (PASW Statistics 18 software).

## Results

### High expression of hBD2 in photo-aged skin

**Fig 1:**
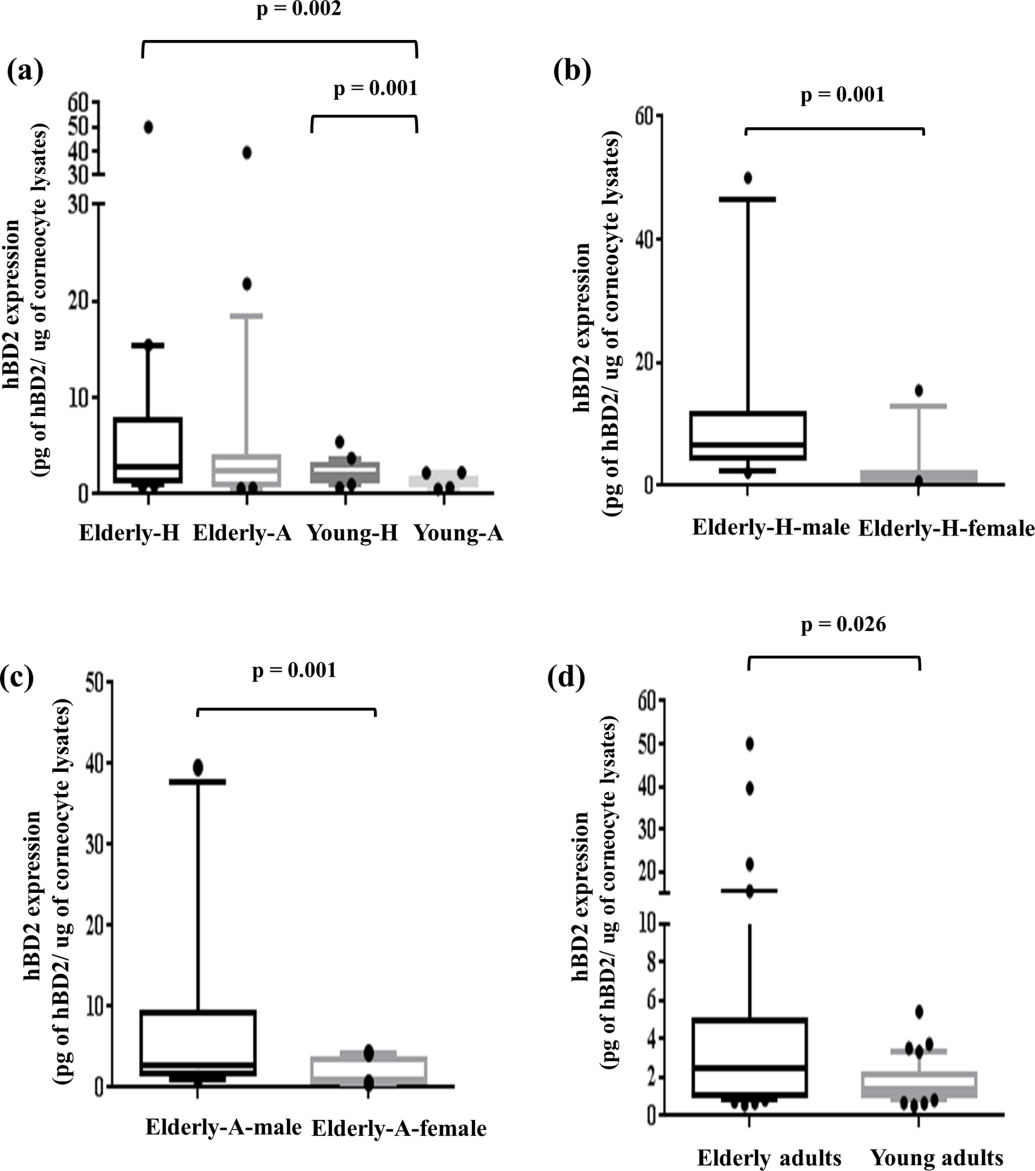
hBD2 expression of human-derived corneocytes affected by UV exposure and aging. Concentrations of hBD2 were determined by ELISA assay. The hBD2 expression of (a) hBD2 expression of total corneocytes between the elderly and young adult groups (n = 42). (b) All groups (elderly adult dorsal hand (Elderly-H), elderly adult abdomen (Elderly-A), young adult dorsal hand (Young-H), and young adult abdomen (Young-A)), elderly adult (c) dorsal hand and (d) abdomen (elderly male n = 10 and female n = 11). In both elderly and young adult groups n = 21. Data are presented as the mean. *p* values for the differences between groups were calculated with the Mann-Whitney test (without outliners).

It has been reported that hBD2 expression is dependent on UV exposure and aging [15, 42]. Therefore, the relationship of cutaneous hBD2 expression between the elderly (64 to 81 years) and young adults (20 to 29 years) was explored in human skin corneocytes collected from sun-exposed (dorsal hand) and unexposed (abdomen) skin. The IRB of the Chung-Ang University hospital (Seoul, Korea) (IRB No. C2015210) approved the protocol for the collection of human-derived corneocytes. Forty-two healthy individuals were recruited, 21 individuals from 20 to 29 years of age (young adults) and 21 individuals from 64 to 81 years of age (elderly adults). All participants were screened based on medical history and the intake of drugs. As expected, a statistical difference between the elderly and young adults was observed (Fig 1A). Also, the elderly and young adults groups were divided into a sun-exposed aged skin (-H) and unexposed skin (-A), respectively, the elderly-H showed higher hBD2 expression than the young-A (*p* = 0.002), and the difference of young-A compared to young-H showed statistical significance (*p* = 0.001). Interestingly, no significance was seen in between the elderly-A and -H, but elderly male adults had higher hBD2 levels than females (Fig 1B and 1C). The young adult group had no statistically significant sexual differences (data not shown).

### Induction of senescence-associated markers by repeated low energy UVB exposure

**Fig 2:**
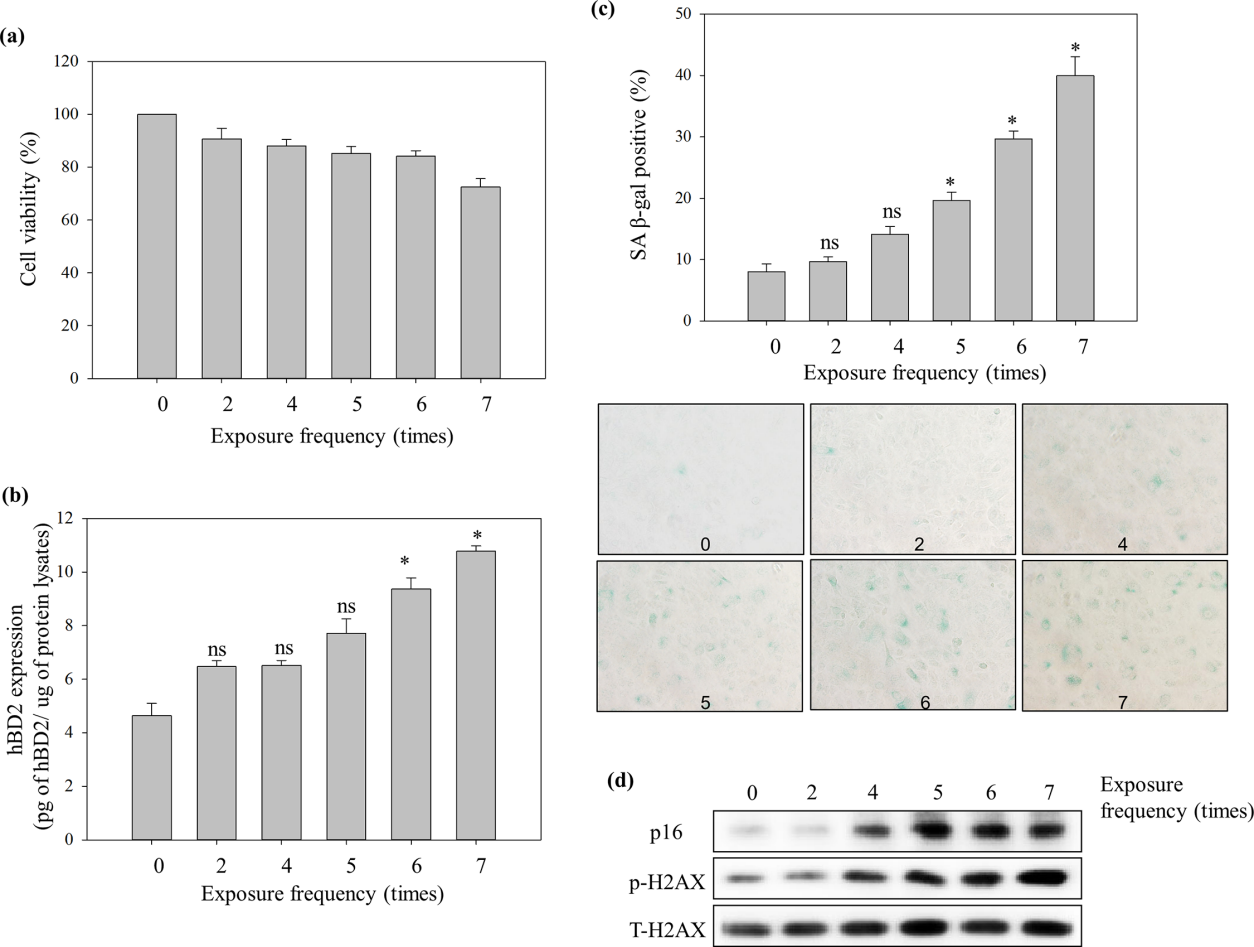
Repeated low energy UVB irradiation-induced increase of SA markers and hBD2 expression in NHEK. NHEK were treated with repeated UVB radiation exposures (5 mJ/cm^2^) at scheduled exposure times (0 to 7 times) and the time interval between exposures was 30 min. (a) Viability, (b) induction of hBD2 expression, (c) β-gal activity and (d) SA-protein markers at 64 h after each repeated exposure to UVB on NHEK. The degree of cell senescence was quantified as the percentage of SA-β-gal positive cells and expressed as a percentage of the 0 exposure group. hBD2 expression was measured using whole cell lysates by ELISA assay. Data are presented as the mean ± SEM of three independent experiments (n = 3). *, *p*<0.05, control *vs*. UVB treatment group. ns, no significance.

Over the last years, studies on aging suggested that p16^INK4a^ is a robust biomarker of intrinsic cellular aging in skin, and is especially involved in UVB-induced senescence [43]. In addition, the histone H2AX has been identified as a component in DNA repair mechanisms and is regarded as a marker to analyze the impact of UVB exposure on the epidermis and is also an indicator of oxidative cell stress in senescent epidermal cells [44, 45]. To determine the optimal condition for the induction of premature senescence, NHEKs were repeatedly irradiated with UVB (5 mJ/cm^2^) with scheduled exposure times (2 to 7 times) with a 30 min time interval between exposures. At 64 h after the last UVB exposure, accumulation of p16^INK4a^ and phosphorylated H2AX protein reached a maximal level after 5 and 7 repeated UVB exposures, respectively (Fig 2D). Also, the percentage of positive cells for SA-β-gal activity increased under the same conditions (Fig 2C). However, cell viability at 64 h after a series of 7 exposures showed sub-cytotoxic stress conditions in the NHEK (Fig 2A). Therefore, a total of 6 exposures to UVB at 5 mJ/cm^2^ were selected as the optimal premature senescence inducible condition.

### The intracellular ROS scavenging effect of adiponectin in repeated low energy UVB-exposed NHEK

**Fig 3:**
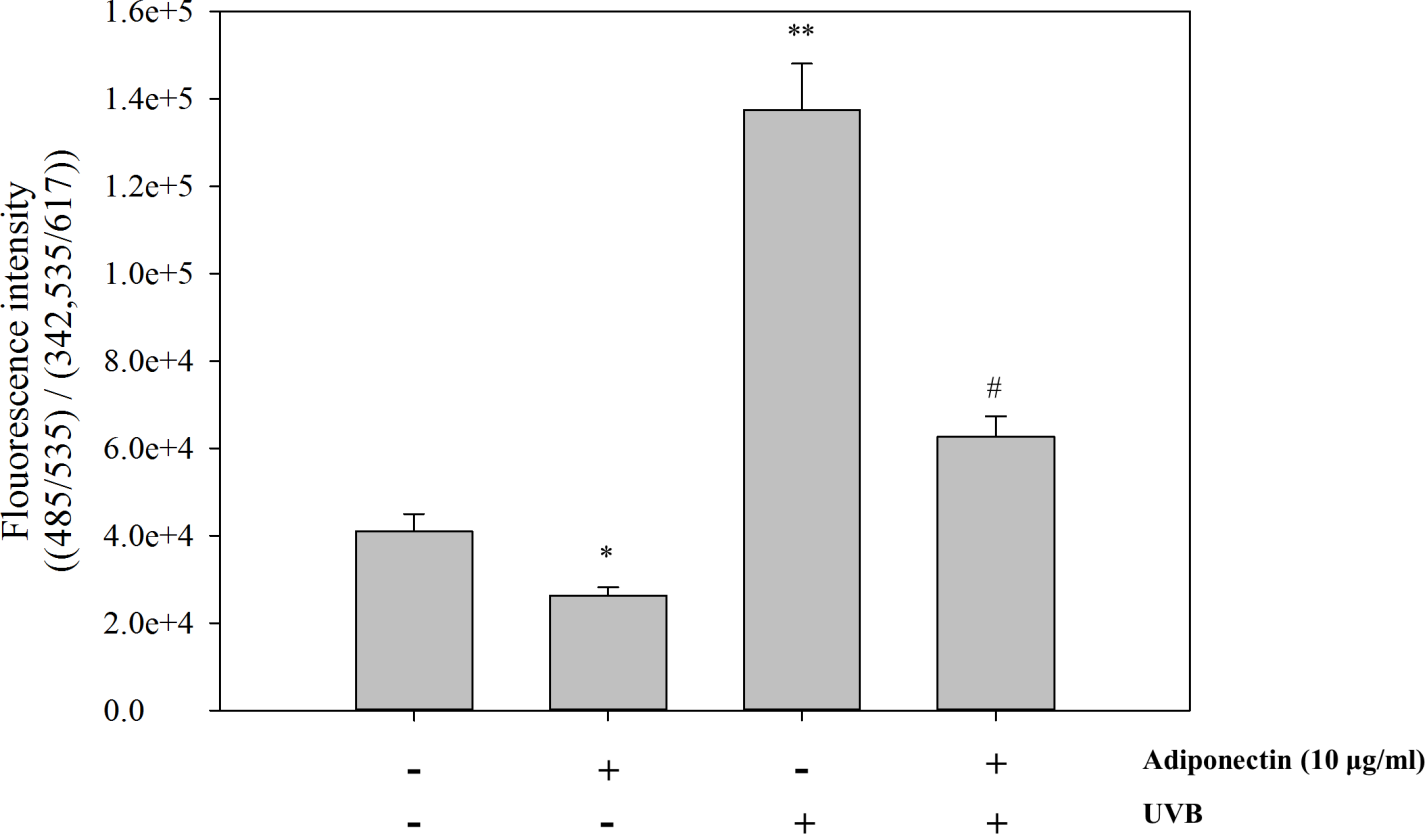
ROS scavenging effect of adiponectin in UVB exposed NHEK. UVB exposure was repeated 6 times and the time interval between exposures was 30 min. Intracellular ROS levels were measured by detecting the fluorescence intensity of the oxidant-sensitive florescent probe DCFH-DA. The florescence intensity was recorded in the presence or absence of adiponectin (10 μg/ml) at 485 nm/ 535 nm. Normalizing of the ROS florescence intensity was calculated using the PI fluorescence measurement intensity ratio. Data are presented as the mean ± SEM of three independent experiments (n = 3). *, *p*<0.05; **, *p*<0.005 *vs*. NC. #, *p*<0.05, *vs*. UVB treated group. ns, no significance.

The dose of adiponectin used in this study was based on the report by Tomizawa, Hattori [46] who showed that pre-incubation with 10 μg/ml adiponectin for 24 h has the maximal ability to suppress pro-inflammatory molecules. To analyze the intracellular ROS scavenging effect of adiponectin, NHEK were exposed with repeated low energy UVB (5 mJ/cm^2^) followed by treatment with 10 μg/ml adiponectin for 24 h. Intracellular ROS levels were measured by detecting the fluorescence intensity of the oxidant-sensitive probe DCFH-DA. As shown in Fig 3, intracellular ROS levels were markedly increased after repeated low energy UVB irradiation exposures (**, *p* = 0.005) and adiponectin significantly reduced UVB induced intracellular ROS levels (#, *p* = 0.011) compared to the control group untreated with adiponectin.

### Effect of adiponectin on the expression of senescence associated markers and hBD2 in premature senescent NHEK

**Fig 4:**
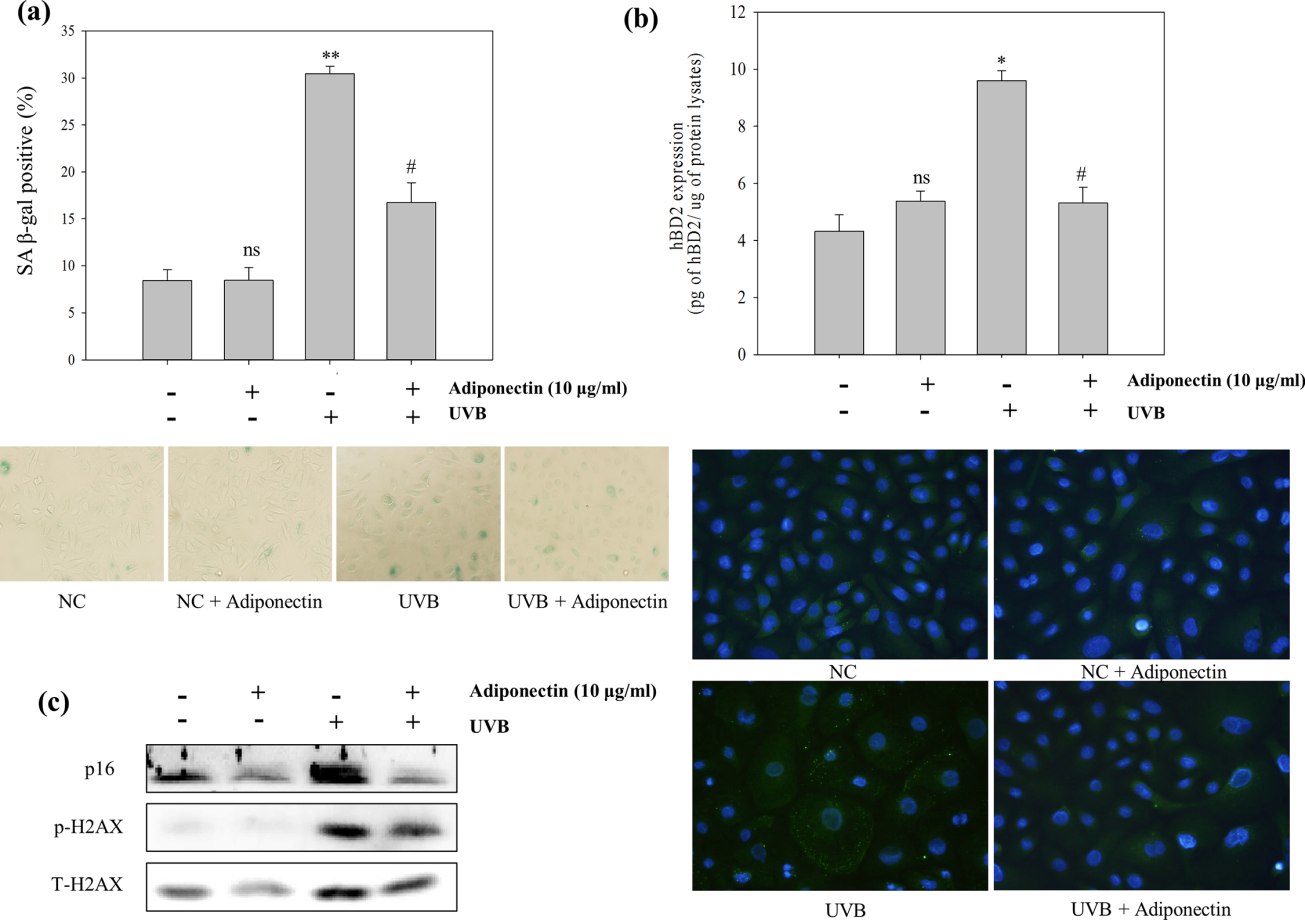
**The protective effect of adiponectin pre-treatment on repeated UVB exposure induced (a) SA-β-gal activity, (b) SA-markers, and (c) hBD2 expression.** NHEK pre-treatment of adiponectin for 24 h, and then treated with 6 repeated UVB exposures. After 64 h, the degree of cell senescence was quantified as the percentage of SA-β-gal positive cells and expressed as a percentage of NC cells. Protein expression levels were analyzed by Western blot. hBD2 expression was measured using the whole cell lysates by ELISA assays (representative fluorescence images of hBD2 expression levels in the presence or absence of adiponectin (10 μg/ml), showed as photographs). Data are presented as the mean ± SEM of three independent experiments (n = 3). *, *p*<0.05; **, *p*<0.005, *vs*. NC. #, *p*<0.05, *vs*. UVB treated group.

NHEK were prepared as described earlier. In premature senescent NHEK (Fig 4A and 4B), adiponectin evidently inhibited SA-β-gal activity (#, *p* = 0.033) and p16^INK4a^ and phosphorylated H2AX protein expression. Moreover, Fig 2B shows an exposure frequency-dependent induction of hBD2 expression with a significant difference compared with NC (*, *p* = 0.023) on premature senescence conditioned NHEK. Indeed, the representative fluorescence images show the morphological changes of premature senescent NHEK and overexpression of hBD2 (Fig 4C). Adiponectin has a statistically significant ability to reduce hBD2 overexpression (#, *p* = 0.039).

### Adiponectin attenuates p38 and JNK/SAPK MAPK signaling in repeated low energy UVB exposed NHEK

**Fig 5:**
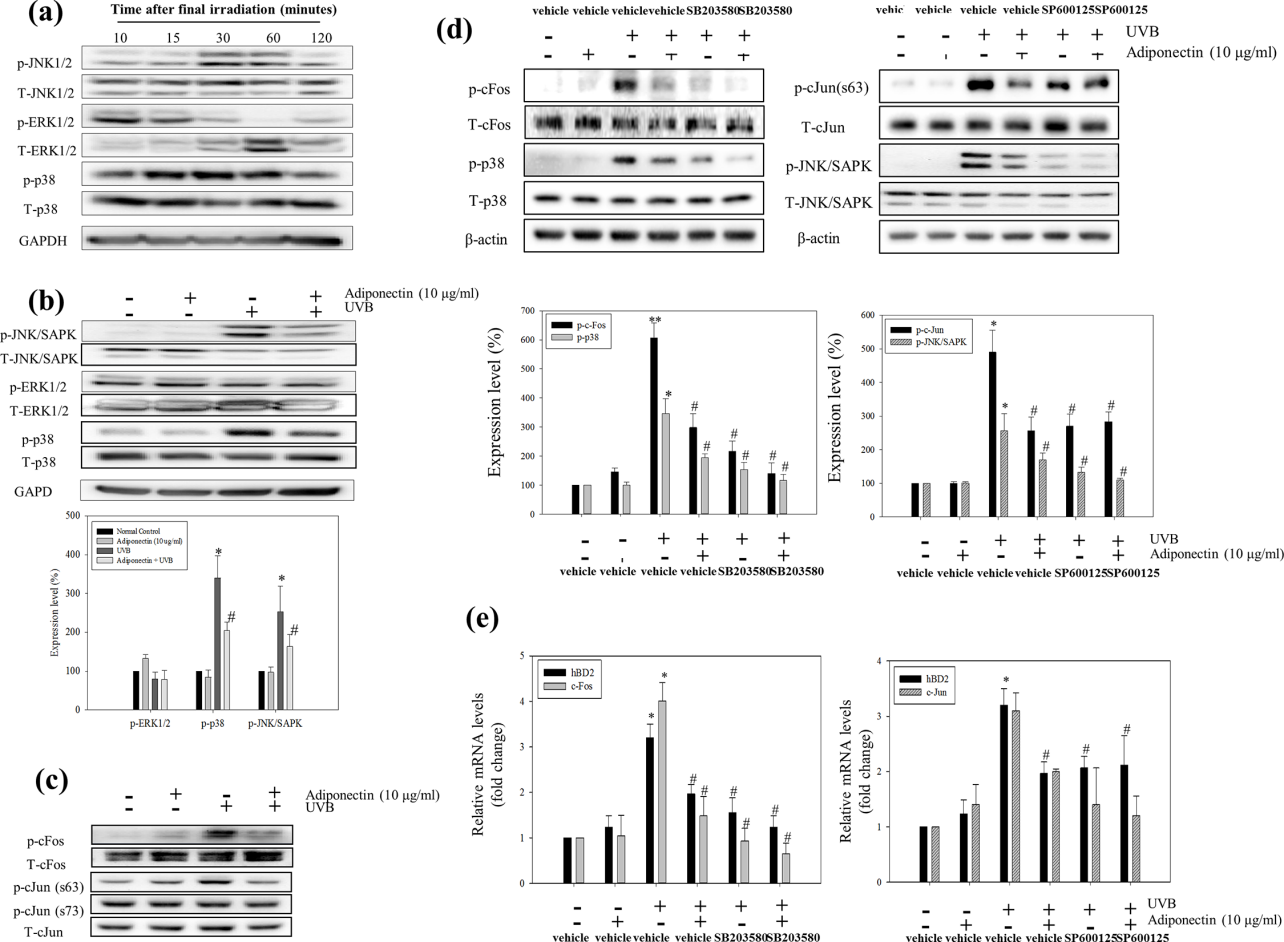
The inhibitory effect of adiponectin on hBD2 expression signaling molecules on repeated UVB exposed NHEK. (a) The time dependent phosphorylation of MAPKs induced by repeated UVB exposure. Adiponectin attenuated the phosphorylation of (b) JNK/SAPK, ERK and p38 MAPK phosphorylation (the relative expression levels were quantified and presented in graphical form) and (c) c-Fos and c-Jun protein expression. The UVB induced upregulation of hBD2 was suppressed through (d) the AP-1 components c-Fos and c-Jun. NHEK were treated with both SP600125 and SB203580 inhibitors before UVB exposure. Protein expression levels were analyzed by Western blot. And the relative (e) mRNA expression of c-Fos, c-Jun and hBD2 are represented in graphical form (fold change compared with NC cells). Data are presented as the mean ± SEM of three independent experiments (n = 3). *, *p*<0.05, *vs*. NC. #, *p*<0.05, *vs*. UVB treated group.

Western blot analysis was used to assess the effect of adiponectin on MAPK signaling pathway molecules. NHEK were prepared as described earlier and protein lysates were harvested at 10 min (ERK1/2) and 30 min (p38 and JNK/SAPK) after a series of 6 UVB exposures. As shown in Fig 5A, repeated UVB exposures induced the phosphorylation of p38 (*, *p* = 0.018) and JNK/SAPK (*, *p* = 0.05) while ERK1/2 protein was constitutively expressed. The UVB-induced phosphorylation levels of p38 and JNK/SAPK were inhibited in the adiponectin pre-treatment groups (Fig 5B).

### hBD2 mRNA expression is dependent on the induction of the AP-1 component c-Fos mRNA expression

As determined in the work of Mineshiba *et al*. (2005), the hBD2 promoter contains an AP-1-binding site. Fig 5C shows that c-Fos and c-Jun protein, components of the transcription factor AP-1, were phosphorylated at 64 h after 6 UVB exposures. To determine whether the c-Fos and c-Jun (serine 63) expression was affected by p38 and JNK/SAPK signaling, the p38 and JNK inhibitors SB203580 (10 μM) and SP600125 (125 nM), respectively, were used. The inhibitors were applied to the cells 1 h before the first UVB exposure and again after the third exposure. Fig 5D shows a reduction of c-Fos and c-Jun phosphorylation by the p38 and JNK/SAPK inhibitors, respectively. Moreover, the phosphorylation of the p38 and c-Fos were most effectively attenuated by applying the SB203580 with adiponectin. Likewise, the induction of hBD2 mRNA expression was observed with the same trend as c-Fos mRNA expression, which was markedly reduced by p38 inhibitor treatment with adiponectin at 30 min after last UVB exposure (Fig 5E). In contrast with c-Fos expression, the c-Jun expression was not parelle with JNK/SAPK expression.

## Discussion

As the first immune barrier component against environmental stress, hBD2 plays a role in cellular immune reactions and provides a link between the innate and adaptive responses against cutaneous infection as a chemotactic factor [3, 8]. However, its uncontrolled expression caused by inflammatory responses leads to immune disorders. Overexpression of hBD2 was found in elderly individuals and UV exposed condition [2, 14–16]. Therefore, the present study explored the relation between photoaging and hBD2 expression. For the investigate the hypothesis, we checked the hBD2 expression on the human derived corneocytes and constructed photoaging *in vitro* model.

There was no significant difference between dorsal hand and abdomen skin in elderly group represented that whether they exposed to sun light or not their hBD2 expression ascended with increasing age and these same trends already revealed in skin fluid and peripheral blood mononuclear cells (PBMCs) [2, 14]. Meanwhile, the possible reasons why elderly adults’ dorsal skin has higher hBD2 expression relative to young adults’ abdomen skin could be due to an accumulation of UV exposure. Interestingly, unlike the young adult group, the elderly adult group showed a statistical difference between genders (Fig 1C and 1D). Based on a recent survey, these results could be explained by the difference in the using frequency of sunscreen and moisturizers (sunscreen (male: female = 51: 96.5), moisturizers (male: female = 27.5: 74.5)). Its cumulative effects throughout the life span might make a difference (Foundation of Korea Cosmetic Industry Institute, 2012). To prove this, a premature senescent NHEK *in vitro* model was constructed by a series of repeated low energy UVB exposures. At one or two exposures to low energy UVB, keratinocytes were able to completely repair the UVB-damaged DNA and continue normal cellular functions. However, increased repeated UVB exposures induced acute oxidative stress in the keratinocytes and impacted the increase of H2AX phosphorylation involved in DNA repair intermediates and accumulation of the tumor suppressor p16^INK4a^, which both have been implicated in cellular senescence. The repeated UVB exposures also showed a high level of SA-β-gal activity (Fig 2). Indeed, morphological changes and overexpression of hBD2 were also observed in senescent keratinocytes. Moreover, the result of clinical research supports the correlation between photoaging and hBD2 expression (Fig 1A).

Recent reports showed the effect of adiponectin secreted from adipose tissue against the inflammatory response and cutaneous disease [35–38]. In the current study, our results show that adiponectin decreases intracellular ROS generation resulting from UVB irradiation. UV irradiation affects calcium influx in keratinocytes, and consequently induces the generation of intracellular ROS [47]. UVB irradiation-induced ROS generation mediated a variety of cellular responses including activation of the MAPK cascade and the induction of DNA damage, resulting in hBD2 overexpression and cellular senescence, respectively. Therefore, as the ROS scavenging effect of adiponectin could result in the prevention of DNA damage mediated by oxidative stress, this novel approach of adiponectin treatment in UV damaged keratinocytes has the possibility to delay cell senescence. Also, all of the *in vitro* results showed that p38 and JNK/SAPK MAPKs that are linked to AP-1 expression were markedly suppressed by adiponectin. It means that the excessive hBD2 expression was attenuated by ROS scavenging which down-regulated of hBD2 trascription in accordance with the decreased of AP-1 expression. And the inhibitor study elucidated c-Fos mRNA expression via p38 activation was the most closely associated with hBD2 mRNA expression.

In conclusion, the present study demonstrates that the effect of adiponectin on scavenging ROS and inhibiting SA-markers in UVB-exposed keratinocytes. The unregulated production of hBD2 by the induction of oxidative stress was attenuated by adiponectin through the suppression of p38 and JNK/SAPK MAPK signaling in UVB-mediated premature senescent NHEK. All these results provide a possibility of adiponectin for anti-photoaging and anti-inflammatory agent in the skin.

## Supporting Information

S1 Fig The UVB induced intracellular ROS was not scavenged by p38 and JNK inhibitors. (TIF)

S2 Fig The repeated UVB irradiation mediated p16 induction was not suppressed by p38 and JNK inhibitors. (TIF)

S3 Fig The protein expression of adiponectin receptor1 was decreased as followed by repeated UVB irradiation. (TIF)

S4 Fig A series of 6 times of repeated UVB exposure was not induced apoptosis in normal human keratinocytes. (TIF)

S5 Fig The adiponectin not only reduced UVB induced cell damage but also promoted cell proliferation in normal human keratinocytes. (TIF)
