# Correction: Adiponectin Suppresses UVB-Induced Premature Senescence and hBD2 Overexpression in Human Keratinocytes

**DOI:** 10.1371/journal.pone.0162738

**Published:** 2016-09-06

**Authors:** MinJeong Kim, Kui Young Park, Mi-Kyung Lee, Taewon Jin, Seong Jun Seo

There are errors in the plus-minus symbols in Fig 5D. The authors have provided a corrected Fig 5 below.

**Fig 5:**
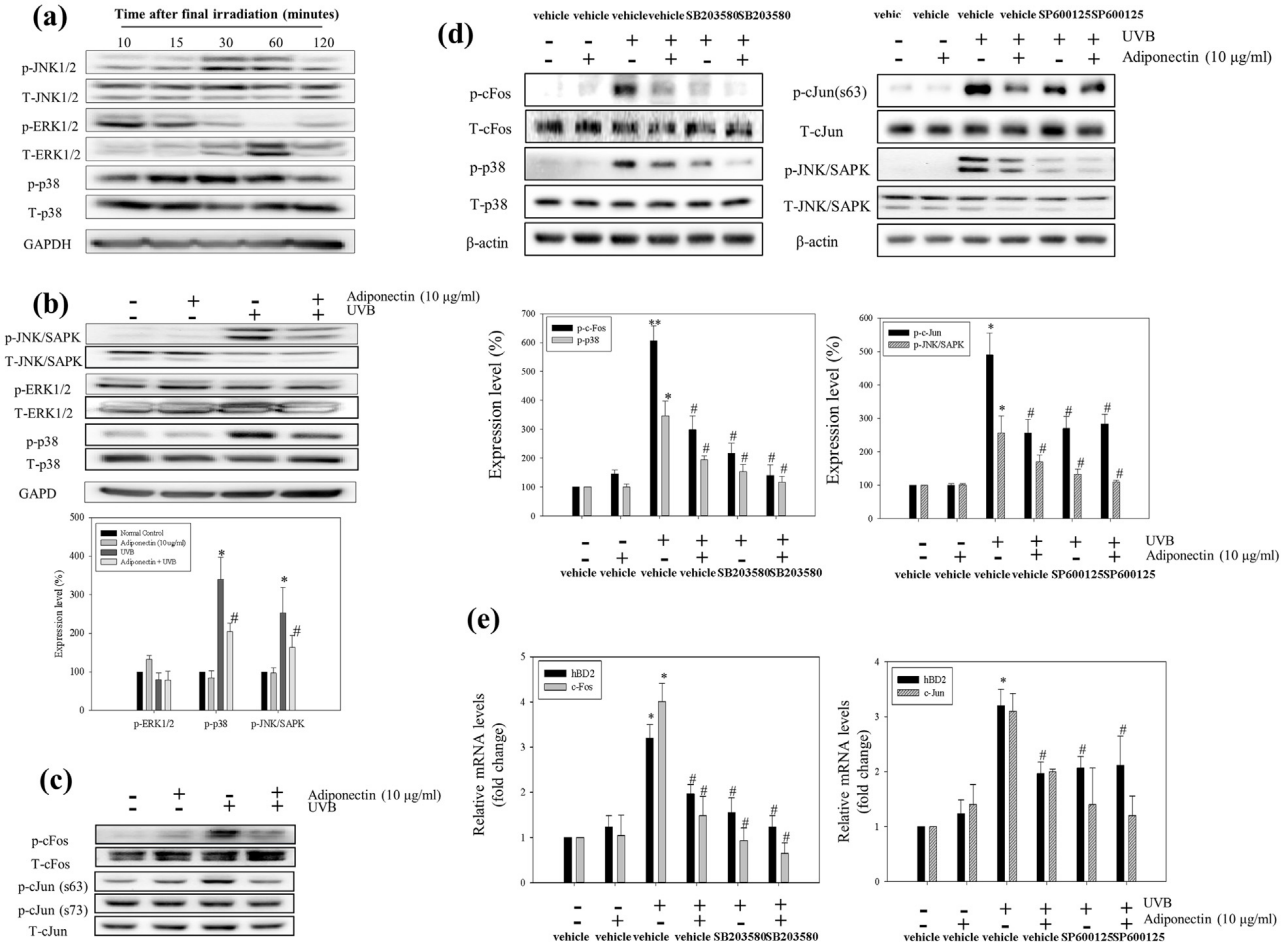
The inhibitory effect of adiponectin on hBD2 expression signaling molecules on repeated UVB exposed NHEK. (a) The time dependent phosphorylation of MAPKs induced by repeated UVB exposure. Adiponectin attenuated the phosphorylation of (b) JNK/SAPK, ERK and p38 MAPK phosphorylation (the relative expression levels were quantified and presented in graphical form) and (c) c-Fos and c-Jun protein expression. The UVB induced upregulation of hBD2 was suppressed through (d) the AP-1 components c-Fos and c-Jun. NHEK were treated with both SP600125 and SB203580 inhibitors before UVB exposure. Protein expression levels were analyzed by Western blot. And the relative (e) mRNA expression of c-Fos, c-Jun and hBD2 are represented in graphical form (fold change compared with NC cells). Data are presented as the mean ± SEM of three independent experiments (n = 3). *, *p*<0.05, *vs*. NC. #, *p*<0.05, *vs*. UVB treated group.
