# Supplementary figures and images for: Adiponectin Suppresses UVB-Induced Premature Senescence and hBD2 Overexpression in Human Keratinocytes
Source: PLoS One. 2016 Aug 15;11(8):e0161247. doi: 10.1371/journal.pone.0161247 (PMC4985158; doi:10.1371/journal.pone.0161247)

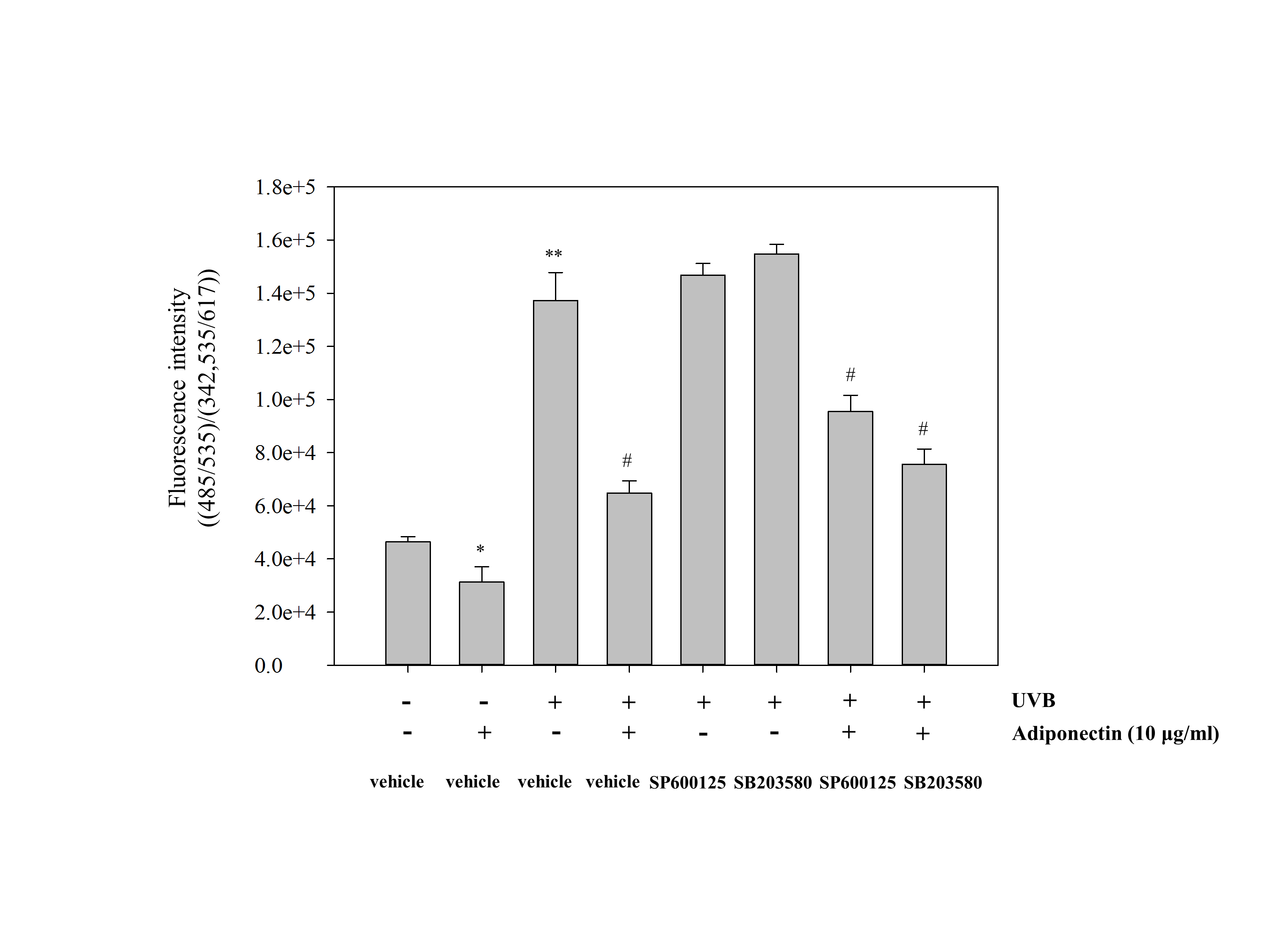

Supplement: S1 Fig — (TIF) [file pone.0161247.s001.tif]

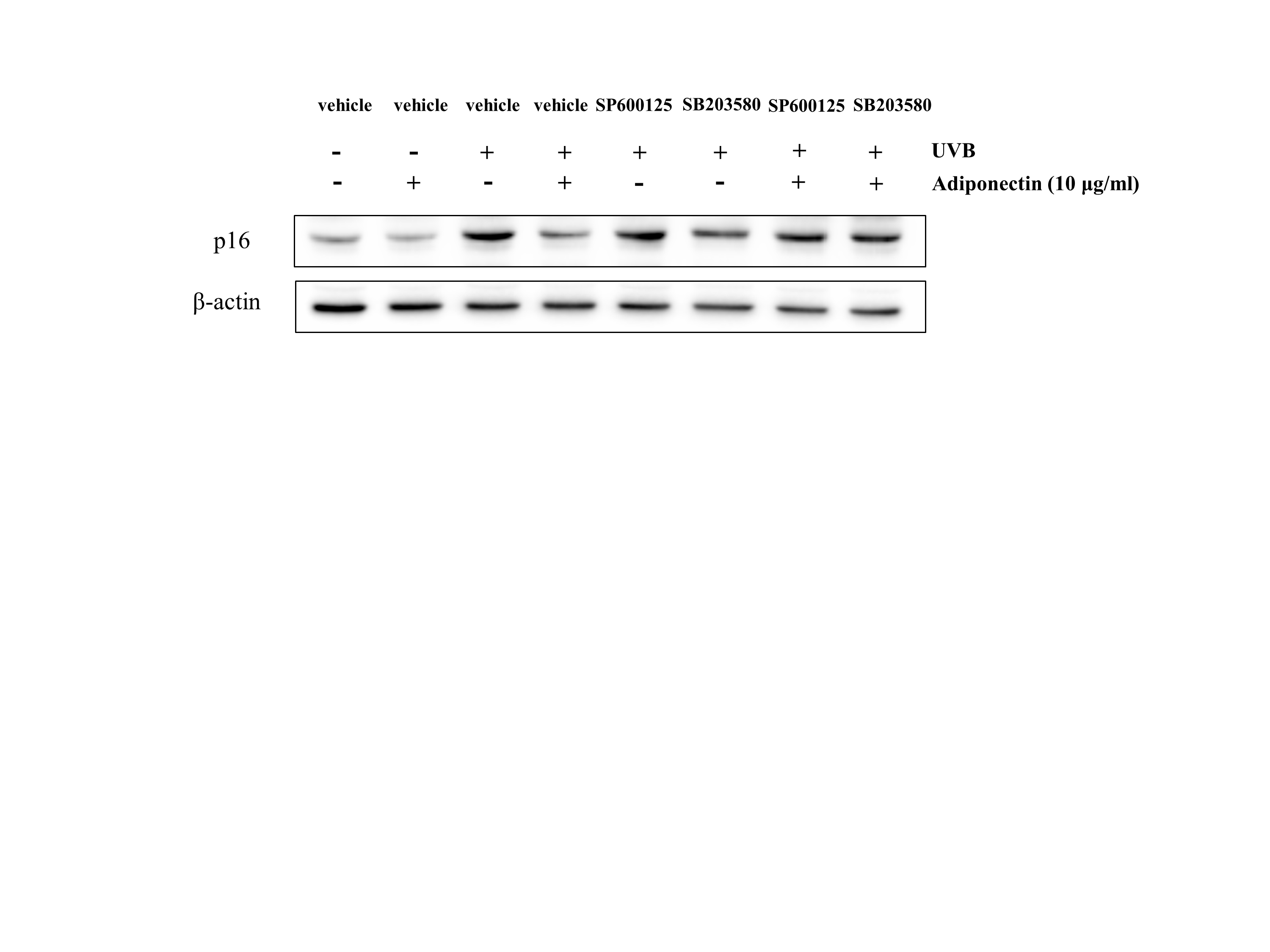

Supplement: S2 Fig — (TIF) [file pone.0161247.s002.tif]

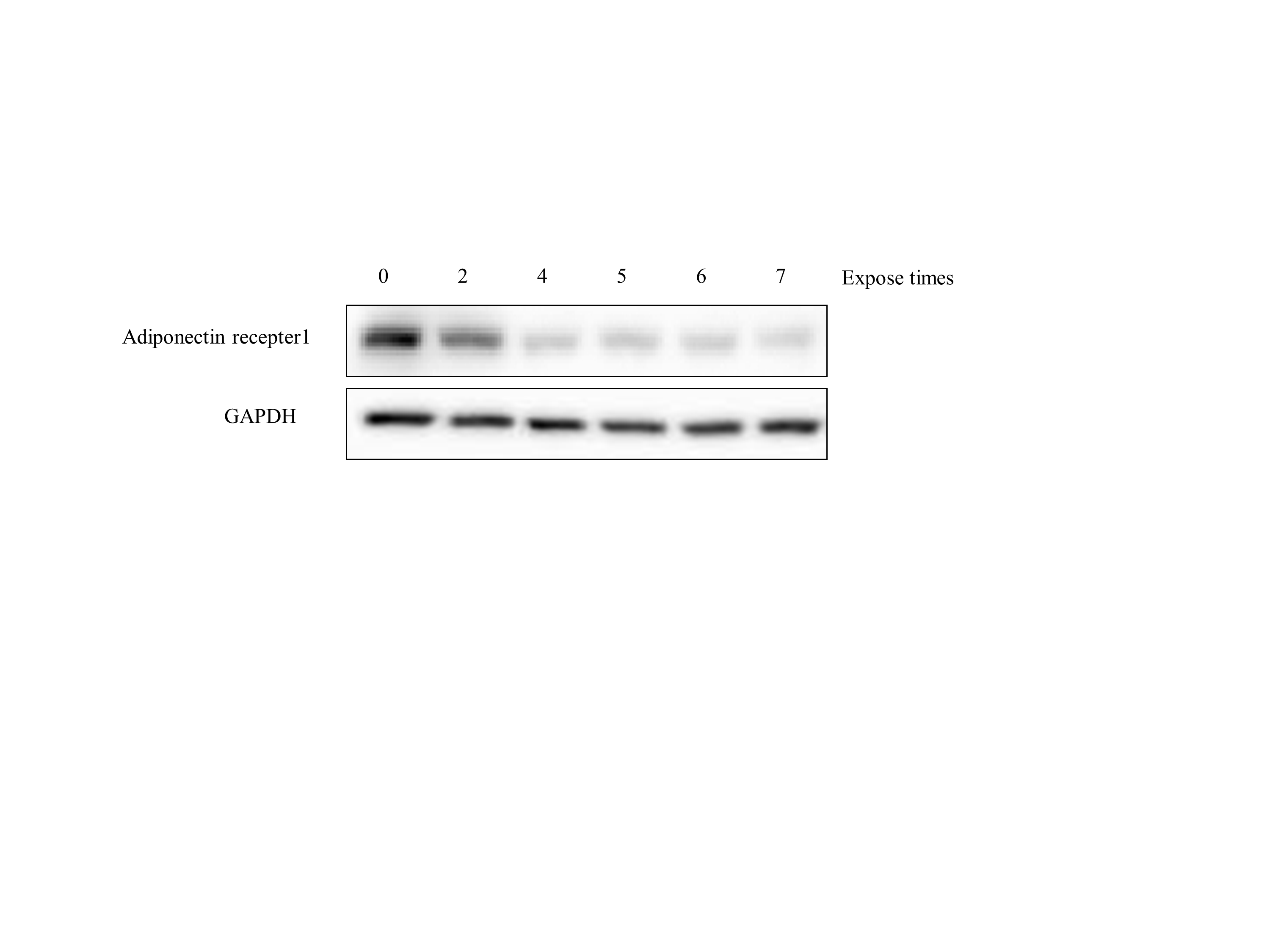

Supplement: S3 Fig — (TIF) [file pone.0161247.s003.tif]

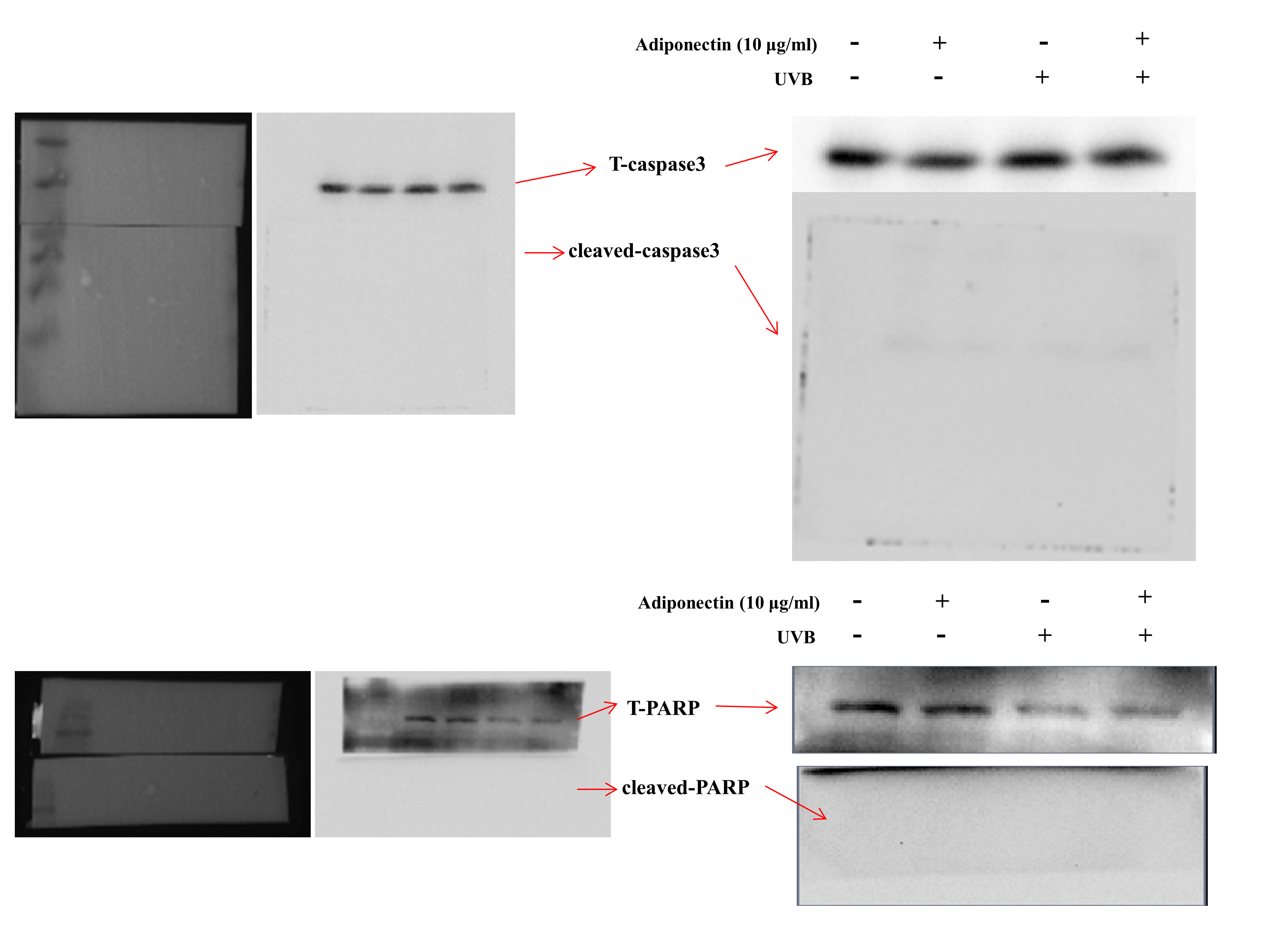

Supplement: S4 Fig — (TIF) [file pone.0161247.s004.tif]

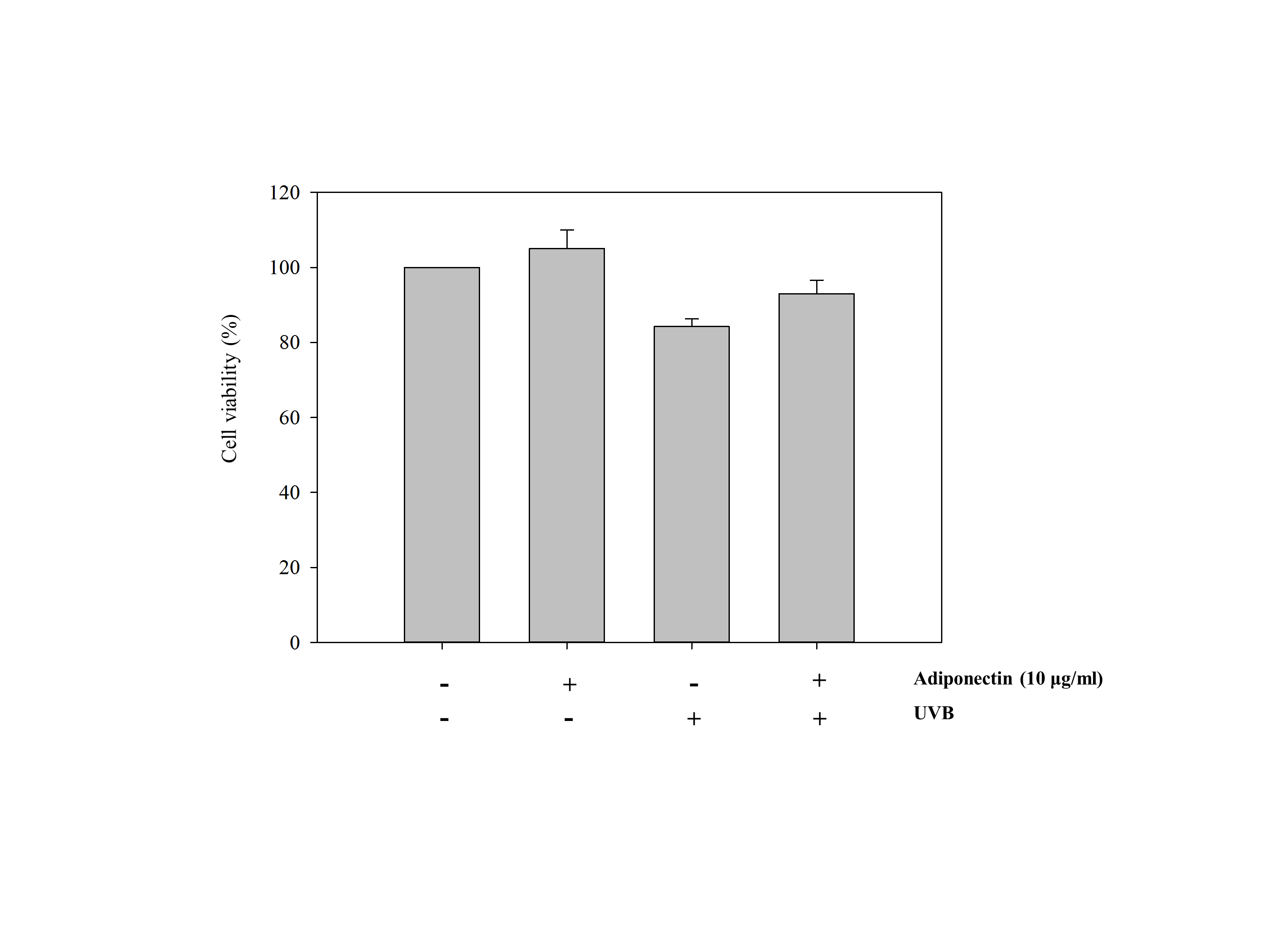

Supplement: S5 Fig — (TIF) [file pone.0161247.s005.tif]
